# Supplementary material for: A Novel Ensemble Learning-Based Computational Method to Predict Protein-Protein Interactions from Protein Primary Sequences
Source: Biology (Basel). 2022 May 19;11(5):775. doi: 10.3390/biology11050775 (PMC9139052; doi:10.3390/biology11050775)
Supplement: Supplementary file 1 [file biology-11-00775-s001.zip › biology-1703166-supplementary.pdf]

In this part, we divide the PPIs dataset into 10 equal parts, 8 of which are subjected to five-fold cross-validation to find the optimal parameters of the rotated forest (feature subset number *K* and decision trees number *L*). The remaining two copies are used as the test set to make predictions. All the final parameters are shown in the following Table S1-S4.

Table S1. The optimal model parameters (*K* and *L*) on the three PPIs datasets.

	K	*L*	validation Acc	validation AUC	Testing Acc	Testing AUC
Yeast	13	21	91.75±0.52%	0.9585±0.0032	90.88	0.9566
Human	9	27	96.10±0.46%	0.9834±0.0038	96.51	0.9828
Oryza sativa	15	25	94.41±0.43%	0.9671±0.0010	94.11	0.9632

Table S2. The prediction results obtained by (train/test/validation) based method on the *Yeast* dataset.

Dataset	Acc (%)	Sens (%)	Spec (%)	PR (%)	MCC (%)	AUC
1	91.15	90.02	92.31	92.30	83.86	0.9533
2	91.64	90.13	93.14	92.88	84.67	0.9614
3	92.27	89.78	94.74	94.43	85.71	0.9602
4	92.31	90.55	94.15	94.18	85.80	0.9576
5	91.37	89.15	93.74	92.82	84.19	0.9598
Average	91.75±0.52	89.92±0.51	93.56±0.93	93.32±0.93	84.85±0.88	0.9585±0.0032
Testing set	91.73	89.93	93.75	93.48	84.82	0.9600

Table S3. The prediction results obtained by (train/test/validation) based method on the *Human* dataset.

Dataset	Acc (%)	Sens (%)	Spec (%)	PR (%)	MCC (%)	AUC
1	96.02	94.84	97.08	96.71	92.32	0.9832
2	95.47	95.11	95.81	95.59	91.34	0.9778
3	96.14	96.60	95.70	95.52	92.57	0.9852
4	96.75	95.19	98.07	97.67	93.66	0.9827
5	96.14	95.15	97.06	96.75	92.56	0.9881
Average	96.10±0.46	95.38±.0.70	96.75±0.99	96.45±0.90	92.49±0.83	0.9834±0.0038
Testing set	95.77	94.58	96.85	95.37	91.87	0.9845

Table S4. The prediction results obtained by (train/test/validation) based method on the *Oryza sativa* dataset.

Dataset	Acc (%)	Sens (%)	Spec (%)	PR (%)	MCC (%)	AUC
1	93.85	94.64	93.12	92.84	88.64	0.9666
2	94.74	94.53	94.84	94.53	90.02	0.9677
3	94.84	84.80	94.59	94.80	90.22	0.9666
4	94.53	93.89	93.90	93.89	89.66	0.9659
5	94.06	95.32	94.97	95.32	88.81	0.9685
Average	94.41±0.43	94.55±0.78	94.28±0.77	94.28±0.99	89.43±0.77	0.9671±0.0010
Testing set	94.11	94.74	93.52	93.24	88.92	0.9632
